# Multiple functions and regulatory network of miR-150 in B lymphocyte-related diseases

**DOI:** 10.3389/fonc.2023.1140813

**Published:** 2023-04-27

**Authors:** Yue-Zi Hu, Qiao Li, Peng-Fei Wang, Xue-Ping Li, Zhao-Lan Hu

**Affiliations:** ^1^ Clinical Laboratory, The Second Affiliated Hospital of Hunan University of Chinese Medicine, Changsha, China; ^2^ Department of Anesthesiology, The Second Affiliated Xiangya Hospital, Central South University, Changsha, China; ^3^ Department of Orthopaedic Surgery, Stanford University School of Medicine, Stanford, CA, United States

**Keywords:** miR-150, B cells, exosomes, autoimmune diseases, B-lymphocyte malignancies

## Abstract

MicroRNAs (miRNAs) play vital roles in the post-transcriptional regulation of gene expression. Previous studies have shown that miR-150 is a crucial regulator of B cell proliferation, differentiation, metabolism, and apoptosis. miR-150 regulates the immune homeostasis during the development of obesity and is aberrantly expressed in multiple B-cell-related malignant tumors. Additionally, the altered expression of MIR-150 is a diagnostic biomarker of various autoimmune diseases. Furthermore, exosome-derived miR-150 is considered as prognostic tool in B cell lymphoma, autoimmune diseases and immune-mediated disorders, suggesting miR-150 plays a vital role in disease onset and progression. In this review, we summarized the miR-150-dependent regulation of B cell function in B cell-related immune diseases.

## 1 Introduction

MicroRNAs (miRNAs) are a type of non-coding RNA molecule that is 20-22nt in length. These RNAs inhibit gene expression by base-pairing targeted mRNAs to avoid ribosomal translation as well as to recruit enzymes to destabilize targeted mRNAs in a ribosome free state (1). Some of miRNAs have multiple target genes, which eventually allows miRNA to regulate complex biological processes and form a complex regulatory network, which is involved in cellular signaling, cross-species variation of gene expression, and co-regulation of transcription factors (2, 3).

MIR-150 is located on human chromosome 19q13.33, downstream of the genes encoding the ribosomal proteins L3a (Rpl13a) and S11 (Rps11) [UCSC Genome Browser (http://genome.ucsc.edu/cqi-bin/hgGateway)]. As an important hematopoietic cell-specific miRNA, miR-150 plays a key role in many hematopoietic lineages, especially lymphocytes. miR-150 accumulates in the lymph nodes, the spleen, and the thymus (4, 5) and is highly expressed in mature B cells and T cells, but does not express in the progenitor cells (6), indicating steady-state levels of miR-150 are the highest during lymphocyte development (4). This stage-specific expression pattern suggests that miR-150 may play a role in lymphocyte development or function (7).

miR-150 is vital in both normal and malignant hematopoietic processes. Studies have demonstrated that miR-150 is a potential target for the treatment of various types of hematopoietic malignancies (8). Low MIR-150 expression was present in Burkitt lymphoma (BL) cell lines, such as Daudi (CVCL_0008), Raji (CVCL_0511), BJAB (CVCL_5711), and Ramos (CVCL_0597). Restoring MIR-150 expression can decrease the proliferation of Daudi and Raji cells. Moreover, ectopic MIR-150 expression impairs the differentiation of pro-B to pre-B stage (9). The expression of MIR-150 in indolent primary cutaneous B-cell lymphoma (10) and chronic lymphocytic leukemia (11) is inversely related to patients’ survival time, helping the prognosis of the disease. Patients with B-cell tumors expressing lower MIR-150 have a worse prognosis and a shorter survival time. This implies that miR-150 is highly relevant in the regulation of B-cell biology regarding physiological conditions and disease states.

This review will summarize the role and mechanisms of miR-150 in regulating B cell biological functions including development, proliferation, differentiation, migration, activation, metabolism, and apoptosis.

## 2 Effect of miR-150 on B lymphocyte biology

The developmental characteristics of B cells are the sequential expression of cell surface markers and the ordered rearrangement of immunoglobulin heavy and light chain gene fragments (12). Through the continuous rearrangement of heavy and light chain loci, B progenitor cells (pro-B) gradually differentiate into precursor B cells (pre-B) and immature B cells expressing membrane-bound immunoglobulin M (IgM). Immature B cells migrate from the bone marrow to the spleen and undergo transitional stages (TR, including T1, T2, and T3) (13, 14). The transitional B cells that enter the splenic follicles are transformed into follicular B cells (15). The spleen, peritoneal cavity, and pleural cavity contain B1 cells (15). B1a cells, a subset of B1 cells, are an important source of serum low-affinity multi-specific IgM antibodies and are related to autoimmunity (16).

The immune response of B cells is regulated by multiple receptor signals and their corresponding molecules, including the B-cell receptor (BCR)-mediated transmembrane signal. After stimulation with an antigen, spleen tyrosine kinase is recruited to BCR phosphorylating tyrosine residues and activating downstream signaling pathways, enzymes, and molecules, such as growth factor receptor bound protein 2-associated binding protein (GAB), and phosphoinositide 3 kinase (PI3K). The activation of these targets then triggers downstream BCR signaling. Upon activation of BCR signaling, multiple response systems are initiated, including the nuclear factor kappa B (NF-κB), the extracellular regulated protein kinase (ERK), and the mitogen-activated protein kinase, and the protein kinase B (AKT) pathways (17). The activations of different enzymes and different pathways lead to the synthesis, assembly, and secretion of various proteins that affect the proliferation, apoptosis, and activation of B cells as well as B cell-related diseases.

**Figure 1:**
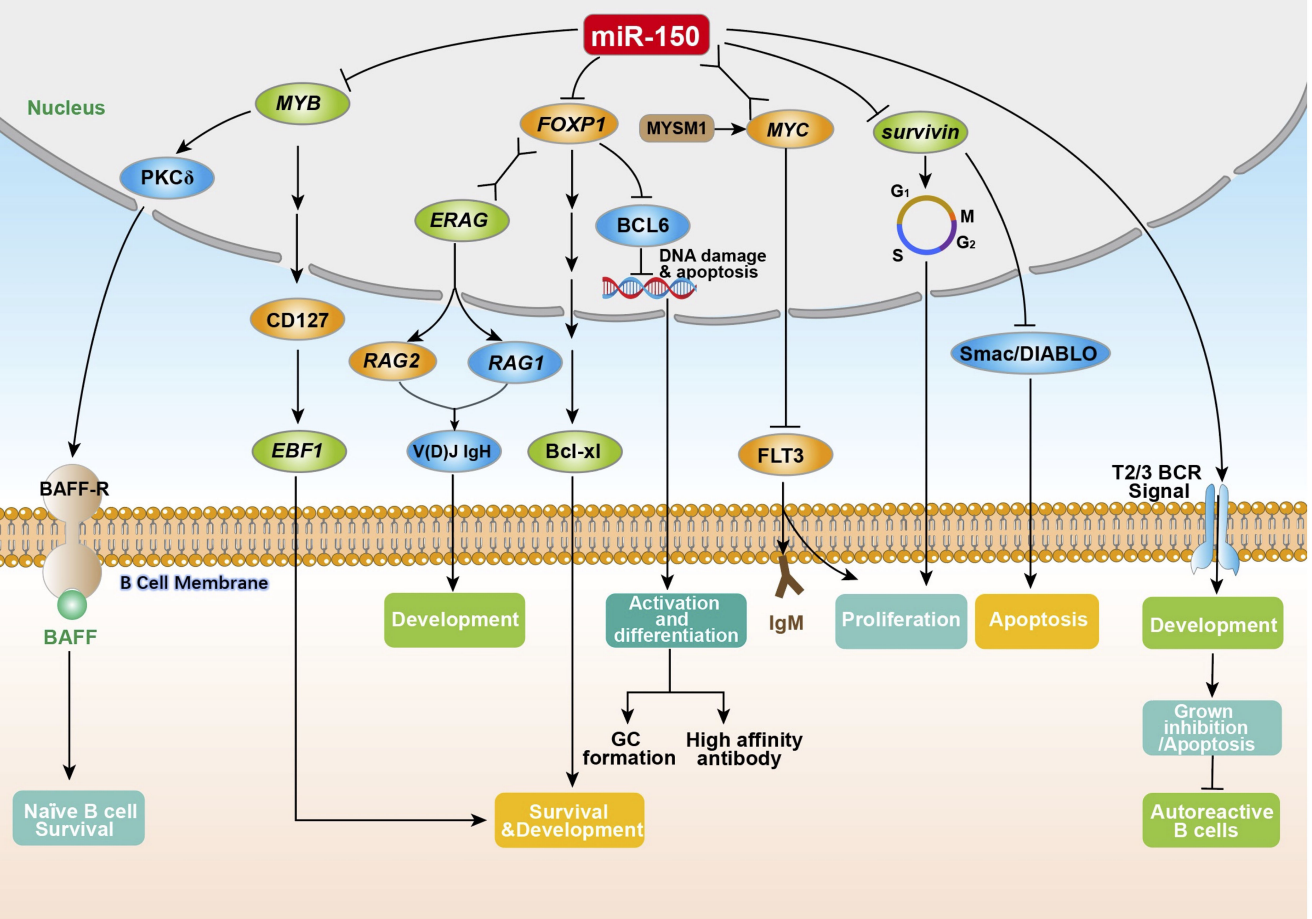
The physiological regulation of miR-150 on B cells. The miR-150 in B cells is directly or indirectly involved in the regulation of key aspects of B cell proliferation, differentiation, development, and antibody secretion by inhibiting the target genes *MYB*, *FOXP1*, *survivin* (participating in the G2/M phase of B cell cycle regulation), and FLT3 (by combining with the *MYC* gene to enhance T2/T3 BCR signaling). *MYB*, MYB proto-oncogene product; PKCδ, nuclear protein kinase cδ; BAFF, B-cell activation factor; BAFF-R, B-cell activation factor receptor; CD127, interleukin-7 receptor alpha-chain; *EBF1*, early B cell factor 1; *FOXP1*, forkhead box factor P1; *ERAG*, Rag enhancer; V(D)J IgH, immunoglobulin heavy chain; *RAG1*, recombination-activating genes 1; *RAG2*, recombination-activating genes 2; Bcl-xl, an anti-apoptotic BCL1 family protein; BCL6, B-cell lymphoma 6; MYSM1, Myb like, SWIRM and MPN domains 1; *MYC*, MYC protooncogene product; FLT3, FMS related receptor tyrosine kinase 3; IgM, immunoglobulin M; Smac/DIABLO, second mitochondria-derived activator of caspase or direct IAP binding protein.

miR-150 enriches in B cells (18), and plays a key role in B cell development and function (4, 7, 19). The ectopic expression of MIR-150 significantly inhibits the differentiation of pro-B cells into pre-B cells as well as affects the development of the entire B lineage (4, 18). Spierings et al. (20) demonstrated that miR-150 regulated the development of immature B-cells by preventing the transition from T1 to T2/3 in peripheral lymphoid organs. Tan et al. (21) found that the expression of MIR-150 was up-regulated within three stages of B cell development including naïve B cells, germinal center (GC) B cells, and memory B cells. Almanza et al. (22) showed that primary B lymphocytes from the spleen of naïve adult mice could synthesize and deliver MIR-150 antisense sequences (anti-microRNA), thus regulating the expression of MIR-150. A large number of studies have shown that miR-150 influences the expression of many proteins, including MYB proto-oncogene product (MYB), forkhead box factor P1 (FOXP1), and FMS related receptor tyrosine kinase 3 (FLT3), as well as regulating B cell survival (21, 23–25) and BCR signaling (26) (**Figure 1**).

### 2.1 *MYB* (HGNC:7545)

MYB is a transcription factor essential for normal lymphocyte development and B cell growth (4, 7). *MYB* knockout in B cells prevents the transition of pro-B cells to pre-B cells (24), consistent with a defect in the lifespan of pre-B cells. This may be because *MYB*-deficient B cells have reduced nuclear protein kinase cδ (PKCδ) levels, thereby inhibiting the expression of the B-cell activation factor receptor (BAFF-R), leading to decreased sensitivity of the B cell to BAFF (the main determinant of naïve B cell survival) (27). Fahl et al. (28) confirmed that *MYB* can upregulate the expression of interleukin-7 receptor alpha-chain (CD127) and enhance the expression of early B cell factor 1 (*EBF1*) (HGNC:3126) promoting the development and survival of pro-B cells. *MYB* is downregulated in immature and mature B cells and upregulated in progenitor lymphatic B cells, pro-B cells, and pre-B cells. The “seed” region of MIR-150 is “complementary” paired with two highly conserved 8-nt sites in the 3’UTR of the *MYB* (7, 29). Ectopic expression of MIR-150 in progenitor B cells downregulated *MYB* levels, and blocked the conversion of pro-B cells to pre-B cells, thereby inhibiting the early development of B cells (4, 6, 7). These studies suggest that miR-150 regulates B cell development and lifespan through the inhibition of *MYB*.

### 2.2 *FOXP1* (HGNC:3823)

The transcription factor FOXP1 is critical for early B cell development (23). FOXP1 belongs to the forkhead box transcription factor family, which is highly expressed in pro-B and pre-B cells (23, 30). *FOXP1* conserved 7-nt locus in its 3’UTR is recognized as a MIR-150 “seed” region (29). FOXP1 is an important regulatory factor of early B cell development and GC response (23, 31). *FOXP1* deficient mice possessed severe B cell development defects during the transition from pro-B to pre-B cells (23, 24); furthermore, mature B cells were severely reduced within the peripheral blood. This recapitulates the phenotype observed in mice ectopically expressing MIR-150 in hematopoietic stem and progenitor cells. FOXP1 is a transcriptional activator of recombination-activating genes *RAG1* (HGNC:9831) and *RAG2* (HGNC:9832), which rearrange the immunoglobulin heavy chain V(D)J IgH, promoting the transition of pro-B to pre-B cells in bone marrow (23). Another study reports that FOXP1 ablation in developing and mature B cells resulted in reduced numbers and frequencies of both follicular and B1 cells impairing antibody production upon T cell-independent immunization *in vivo*. *FOXP1*-deficient B cells are prone to apoptosis even though they exhibit an increased capacity to proliferate. The transcriptional analysis further demonstrated that overexpression of B-cell lymphoma 2 gene (*BCL2*) (HGNC:990) rescued the survival defect of *FOXP1*-deficient mature B cells *in vivo* and restored the numbers of peripheral B cells (32). *FOXP1* may play antagonistic roles in regulating the GC response. *FOXP1* is downregulated in mature GC B cells and is inversely correlated with BCL6 (31). Together, *FOXP1* is a target of MIR-150 that controls the proliferation, differentiation, and survival of B cells.

### 2.3 *FLT3* (HGNC:3765)

FLT3 is a membrane-bound receptor tyrosine kinase that participates in the proliferation, differentiation, and apoptosis of hematopoietic cells (33). Jiang et al. (34) found that miR-150 directly targets the 3’UTR of *FLT3*. In B1a cells, Myb like, SWIRM, and MPN domains 1 (MYSM1) recruits the transcription factor MYC to the MIR-150 promoter and stimulates MIR-150 transcription, thereby, reducing the expression of *FLT3* in B1a cells, leading to a reduction in proliferation and cell surface IgM levels (25). This study reveals the importance of the MYSM1/miR-150/FLT3 pathway in regulating the proliferation of B1a cells.

### 2.4 *Survivin* (HGNC:593)

Survivin is one of the main inhibitors in the apoptosis (IAP) family. It regulates B cell response to mitotic stimulation and cell cycle progression. Survivin maintains the humoral response of B cells (35) by sustaining the proliferation of B1 and B2 cells (36) and inhibiting their apoptosis by antagonizing the pro-apoptotic protein second mitochondria-derived activator of caspase or direct IAP binding protein (Smac/DIABLO), a mitochondrial protein (37). The 3’UTR of *survivin* contains a 6-nt locus complementary to the MIR-150 “seed” (37). A study of GC B cells in normal tonsils found that MIR-150 expression is inversely correlated with survivin. Transfection of synthetic miR-150 inhibited the expression of *survivin* in the DG75 cell line, indicating that miR-150 regulates B cell survival by regulating the expression of *survivin* directly or indirectly (21).

### 2.5 BCR

The fate of B cells depends on the balance between survival and death signals induced by BCR. BCR signaling may lead to different biological outcomes, depending on the signal strength and duration of BCR, the differentiation stage of the B cells, and whether there is a common stimulus signal (38). Upon activating BCR signaling, immature B cells will stagnate and undergo apoptosis while mature B cells will proliferate (39). Kluiver et al. (26) found that high MIR-150 expression in the T2/T3 phase of transitional B cells activates BCR signaling, resulting in the BCR-induced inhibition of the growth and/or apoptosis of transitional B cells. Conversely, decreased miR-150 in transitional B cells weakens the BCR signaling and prevents BCR-induced apoptosis; thus, promoting the outgrowth of self-autoreactive B cells, leading to autoimmune diseases or even lymphomas.

## 3 miR-150 modulation in B lymphocyte-related diseases

### 3.1 The role of miR-150 in obesity-related diseases

Diabetes mellitus (DM) is the collective term for heterogeneous metabolic disorders whose main finding is chronic hyperglycemia, including type-1 diabetes mellitus (T1DM) and type-2 diabetes mellitus (T2DM). Patients with T1DM exhibit decreased expression levels of MIR-150 in peripheral blood mononuclear cells (PBMCs) compared to healthy control subjects and T2DM patients (40). NF-κB inhibited the apoptosis of pancreatic β cells and islet inflammation by up-regulating the MIR-150 and down-regulating *p53* (HGNC:11998) up-regulated modulator of apoptosis, therefore, preventing the occurrence and development of T1DM (41). However, miR-150 levels and functions differ in two types of DM.

**Figure 2:**
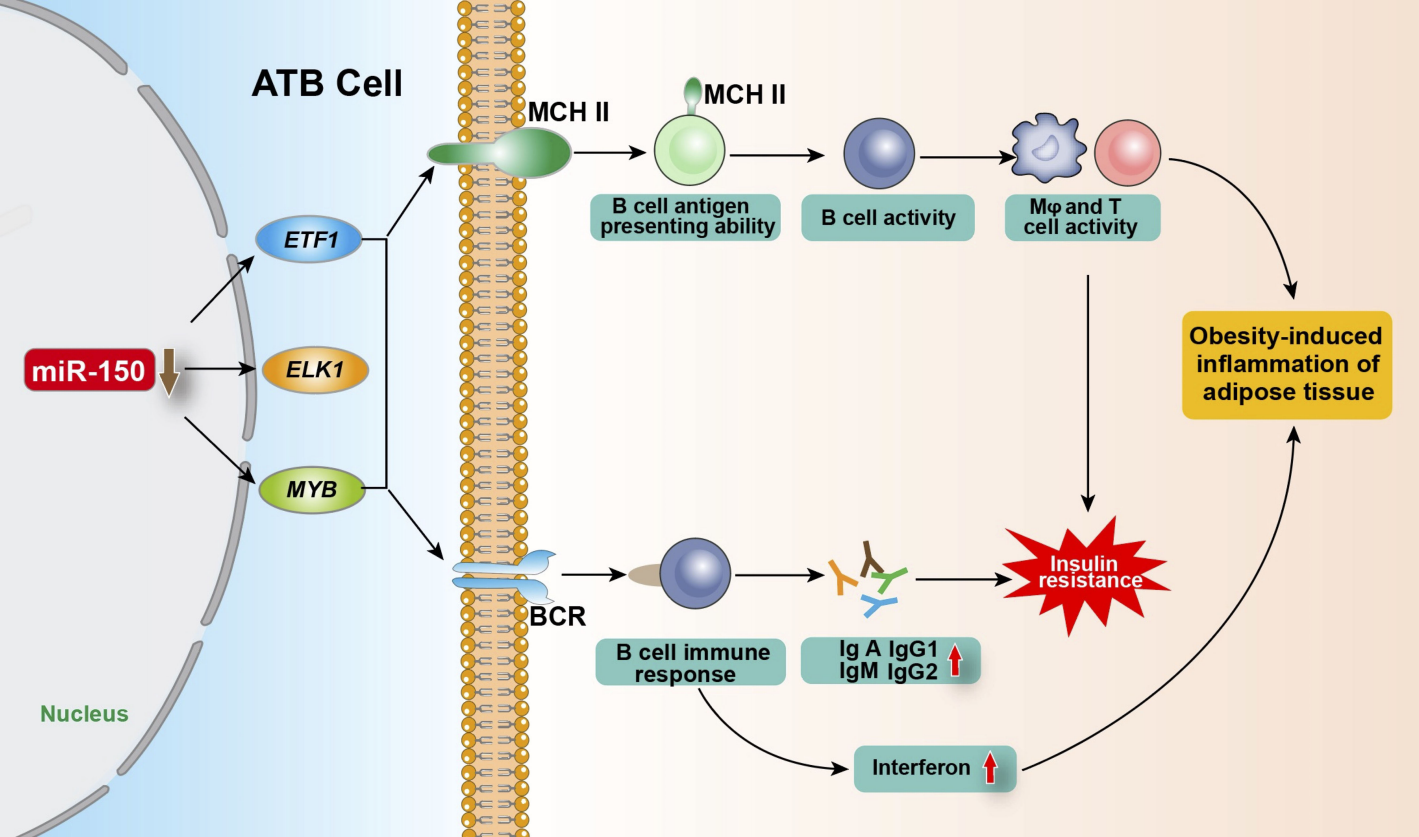
Regulation of miR-150 in adipose tissue B cells on obesity-induced adipose tissue inflammation and insulin resistance. After being stimulated by antigen, ATB regulate the expression of target genes *ETF1*, *ELK1* and *MYB* by reducing miR-150 level, and enhancing the proportion of B cells with MHC II on the cell membrane and the number of MHC II expressed on the B cell membrane so as to improve its antigen presentation ability and its activity as well as enhancing the BCR signaling pathway and immune response, promoting the production of various types of immunoglobulins and interferon, thereby regulating obesity-induced fat tissue inflammation and insulin resistance. ATB, adipose tissue B cells; *ELK1*, ETS transcription factor; *ETF1*, eukaryotic translation termination factor 1; *MYB*, MYB proto-oncogene; MHC II, major histocompatibility complex II; Mφ, Macrophages; BCR, B-cell receptor.

Meta inflammation is involved in the pathogenesis of obesity-related diseases, including T2DM and cardiovascular diseases (42–45). During the occurrence of obesity, the absolute number and relative proportion of adipose tissue B cells (ATB) in visceral stromal cells increase significantly. ATBs are antigen-presenting cells account for more than 20% of the stromal cell population within the fat tissue of obese individuals (46, 47). Ying et al. (18) found that miR-150 controls ATB function by inhibiting the expression of BCR signaling-associated genes including *ELK1* (ETS transcription factor) (HGNC:3321), *ETF1* (eukaryotic translation termination factor 1) (HGNC:3477), and *MYB*, and by changing the surface characteristics of the major histocompatibility complex II (MHC II), thereby regulating insulin resistance, obesity-induced inflammation, and the glucose tolerance of adipose tissue. MIR-150 knockout in ATBs increased the proportion and number of ATB cells, MHC II expression, and the antigen presentation ability of ATB cells, leading to the enhanced activity of T cells or macrophages (Mφ) and fat-induced inflammation and insulin resistance. Knockout of MIR-150 also enhanced the expression of inflammatory cytokines such as interferon γ in adipose tissue. In addition, upon the initiation of B cell response, the aberrantly downregulated MIR-150 increases immunoglobulins production, playing a critical role in obesity-induced insulin resistance (18, 46). Consistently, Xiao et al. (7) demonstrates that MIR-150 knockout animals had a multi-fold increase in IgA, IgG1, IgG2, and IgM serum levels. The elevated serum immunoglobulin levels in MIR-150-deficient animals are likely due to the increased response of follicular B cells. Recently, He et al. (48) found that in T2DM intestinal damp-heat syndrome patients, compared to healthy people, the exosomal miR-150 was significantly upregulated, and the total cholesterol and triglyceride contents of diabetic patients were positively correlated with exosomal miR-150 expression. Collectively, miR-150 plays a key role in ATB function by regulating immune homeostasis within adipose tissue (**Figure 2**).

### 3.2 The effect of miR-150 on B cell lymphoma

miR-150 is one of the most abundant miRNAs within three mature B cell subsets: naïve, GC, and memory B cells (21). miR-150 is important in regulating the expression of genes associated with BCR signaling (7, 11, 49–52). Recently, miR-150 started to receive increasing attention due to its tumor suppressive role in hematological malignancies (53). MIR-150 is downregulated in several B-lymphocyte malignancies (49, 54–57), such as diffuse large B-cell lymphoma (DLBCL) (55), mantle cell lymphoma (MCL) (54), BL (9) and aggressive chronic lymphocytic leukemia (CLL) (11). Besides, the MIR-150 is highly expressed in lymphocyte-derived exosomes and is easily detectable and significantly enriched in exosomes circulating in human blood (58). Exosome-encapsulated miR-150 is being suggested as a new class novel biomarker as diagnostic and predictive markers in the development of B cell malignancies (59, 60). Abnormal expression of MIR-150 results in dysfunction of important genes that perform the function in the survival, proliferation, and aggressiveness in B cell malignancies (61) (**Table 1**).

**Table 1:** The effects of miR-150 on B lymphocytes malignant tumors.

Disease	Samples	miR-150	Putative targets	Effect on B cells	Impact on disease	Reference
FL	Lymph node	↓	*FOXP1*	Apoptosis↓	Promote	(49, 62–65)
			*SLC2A1*	Glucose metabolism↑		(66, 67)
CLL	Peripheral B cells	↓ (poor prognosis)	*FOXP1*	Proliferation↑ Apoptosis↓	Promote	(11, 23, 31)
			*GAB1*	Cross-linking response to BCR↑		(11, 68)
			CXCR4	Migration↑		(69)
	CLL-derived exosomes	↑	BCR	NA	Promote	(60, 70, 71)
			Hematopoiesis			
DLBCL	Peripheral B cells	↓	*FOXP1*	Growth↑ Apoptosis↓	Promote	(55, 57, 62, 72–74)
BL (EBV-positive)	Cell lines	↓	*Survivin*	Proliferation↑	Promote	(9, 75)
			*MYB*	Differentiation↓		(9)
MALT (Conjunctiva)	Lymphoma Tissue	↑	*Cbl-b*	Proliferation↑	Promote	(76)
MALT (Gastric)	Mice stomachs	↑	*EGR2*	Apoptosis↓		(77, 78)
MCL	Lymph node	↓	NA	NA	NA	(54, 56, 79)

FL, follicular lymphoma; CLL, chronic lymphocytic leukemia; DLBCL, diffuse large B-cell lymphoma; BL (EBV-positive), Burkitt lymphoma (Epstein-Barr virus-positive); MALT, mucosa-associated lymphoid tissue; MCL, mantle cell lymphoma; *FOXP1*, forkhead box factor P1; *SLC2A1*, solute carrier family 2 member 1; *GAB1*, growth factor receptor-bound protein 2-associated binding protein 1; CXCR4, Chemokine receptor 4; BCR, B-cell receptor; *MYB*, MYB proto-oncogene product; *Cbl-b*, Casitas B-lineage lymphoma proto-oncogene b; *EGR2*, early growth response 2; NA, Not available. ↑, increase; ↓, decrease.

#### 3.2.1 miR-150 in FL

FL and a high-grade transformed follicular lymphoma (tFL) are mediated by the *MYC* (HGNC:7553)*/*MIR-150*/FOXP1* axis. MYC in tFL B cells binds to the promoter of MIR-150 and inhibits its expression in tFL patients (49). The low miR-150 levels in tFL enhance the expression of *FOXP1*. A low level of miR-150 and a high level of FOXP1 are associated with a short overall survival rate (49). These studies indicate that the *MYC/*MIR-150*/FOXP1* axis determines the invasiveness and transformation of untransformed FL B cells (49).

In contrast to normal differentiated cells, which rely primarily on mitochondrial oxidative phosphorylation to generate the energy needed for cellular processes, most cancer cells rely on aerobic glycolysis, a phenomenon termed “the Warburg effect” (80). Even in the context of the aerobic environment, the metabolism of cancer cells leads to a high rate of glucose consumption through glycolysis and the release of lactic acid (81). Tumor cells take up glucose through the glucose transporter 1 (GLUT1) to maintain their anabolic metabolism, growth, and reproduction. King et al. (82) identified the GLUT1 encoding transcript solute carrier family 2 member 1 (*SLC2A1*) (HGNC:11005) as a target of MIR-150, connecting miR-150 with the modulation of glucose uptake. Magi et al. (66) confirmed that *SLC2A1* is overexpressed in FL cells, involved in the control of glucose metabolism, and is associated with FL transformation (67). The downregulation of MIR-150 may promote FL transformation by enhancing glucose metabolism through the upregulation of *SLC2A1*/GLUT1 in FL tumor cells.

#### 3.2.2 miR-150 in CLL

CLL is a mature B-cell malignant tumor characterized by CD5^+^ B-cell clonal accumulation in peripheral blood, bone marrow, and secondary lymphoid organs (83, 84). Low miR-150 levels are associated with a poor prognosis in CLL, possibly due to dysregulated BCR signaling (11). miR-150 influences BCR signaling in CLL by regulating the expression of *growth factor receptor-bound protein 2-associated binding protein 1 (GAB1)* (HGNC:4066) and *FOXP1* (11). Silencing *FOXP1* in B cells down-regulates basal and anti-μ-induced phosphorylated AKT levels (85). Furthermore, FOXP1 is a transcription factor that controls the maturation of B cells by promoting the expression of genes required for the rearrangement of immunoglobulin sub-genes in mature B cells (23). Therefore, silencing *FOXP1* reduces its responsiveness to BCR stimulation (11). In addition, as an important regulator of B cell activation, FOXP1 positively regulates NF-κB signaling in malignant B cells. GAB1 up-regulates the sensitivity of B cells to anti-μ-induced AKT phosphorylation. It is also an adaptor molecule that recruits PI3K to the B cell membrane after sIg connection, activating AKT to enhance the BCR signal. Silencing *GAB1* in B cell lines affects the magnitude of their cross-linking response to BCR directly (68). Low levels of miR-150 in CLL patients increase BCR signaling by increasing the levels of FOXP1 and GAB1 (11), resulting in the increased invasiveness of CLL B cells. However, miR-150 was found upregulated in CLL in both CD5^-^ and CD5^+^ B cells and in 70kDa zeta chain-related protein-positive and IgVH mutated patients in certain cases (11, 59, 86), and it plays a critical role in the hematopoiesis process, especially in the differentiation and development of lymphoid lineage (8). Despite the large amount of circulating miR-150, free or associated with serum proteins, the CLL-derived exosomes can assemble miR-150 in order to protect it from the RNase and thus sustain its pro-tumorigenic action (60, 70). Moreover, miR-150 present in CLL exosomes are transferred to target cells and functionally active (87, 88). Additionally, expression of MIR-150 in exosomes was significantly elevated in CLL-derived exosomes compare to normal B cells and it further increased under α-IgM stimulation. The data implicates the BCR signaling pathway in the control of CLL exosome secretion and its conveyance of the disease-relevant miR-150 (71). All the data above suggests that the regulation of miR-150 in CLL development is beyond BCR signaling and extremely complex.

miR-150 also targets Chemokine receptor 4 (CXCR4) which regulates the migration of mature B cells to secondary lymphoid tissue (89). Studies have shown that ischemia downregulates MIR-150 in bone marrow-derived mononuclear cells, thereby upregulating CXCR4, leading to enhanced cell migration (89). Similarly, the low expression of MIR-150 upregulates CXCR4 in CLL, enhancing its response to Chemokine ligand 12 (CXCL12). This leads to an increase in AKT and ERK signals, malignant B cells infiltration into lymph nodes, and resistance of tumor cells to spontaneous or drug-induced apoptosis (90–93). These studies revealed a miR150-dependent survival pathway of tumor cells, which partly explains their unresponsiveness to conventional chemotherapy.

#### 3.2.3 miR-150 in DLBCL

Compared with memory B cells, miR-150 is much lower in other peripheral B-cell subpopulations, such as naïve B cells and centroblasts in DLBCL patients (55). Low MIR-150 expression is associated with poor clinical outcome predictions in patients with primary gastrointestinal DLBCL (57). Reduced miR-150 causes *FOXP1* upregulation, promoting the growth and survival of B cells in DLBCL by enhancing BCR and NF-κB signaling (62–65). The upregulation of *FOXP1* can promote CBP (β-catenin acetylation through cAMP responsive element binding protein (CREB) binding protein), and enhance Wnt signaling, thereby promoting cell growth (72). The high expression of *FOXP1* is associated with poor clinical prognosis (73, 74). *FOXP1* overexpression in DLBCL cells and human primary B cells inhibit pro-apoptotic genes including *BIK* (BCL2 interacting killer) (HGNC:1051), *EAF2* (ELL (eleven-nineteen lysine-rich leukemia gene) -associated factor 2) (HGNC:23115), and *HRK* (Harakiri) (HGNC:5185), and cooperates with NF-κB to promote B cell survival (62). Therefore, miR-150 promotes the growth and survival of DLBCL cells by regulating the expression of *FOXP1*, and has a clinical value as a prognostic biomarker. DLBCL exosomes promote cell proliferation, migration and angiogenesis *in vitro* (94), and they could induce the transformation of macrophages to a protumor M2-like phenotype, and block the drug-induced apoptosis of DLBCL cells (95).

#### 3.2.4 miR-150 in BL

BL is a highly invasive B-cell lymphoma that consists of two forms of BL differing in Epstein-Barr virus (EBV) infection status, EBV-negative BL and EBV-positive BL. Four types of BL cell lines have been identified: BJAB and Ramos cells are derived from EBV-negative GC B cells, and Daudi and Raji cells are derived from EBV-positive GC B cells. The expression level of MIR-150 is extremely low in these four types of BL cell lines (9). A low miR-150 level leads to a high expression level of *survivin* and *MYB*, promoting tumor cell proliferation and preventing differentiation (75). When the MIR-150 expression is increased in Daudi and Raji cells, cell proliferation is significantly reduced while their differentiation is induced (9, 75). These findings suggest that miR-150 can regulate the expression of *survivin* and *MYB* and affect the proliferation and differentiation of EBV-positive BL cells. Additionally, B cell–derived exosomes released from EBV infected B cells are able to deliver their content to B cells, and thereby, influence B cell biology (96). B cell lymphoma-derived exosomes upregulated inhibitory receptors PD-1 (programmed cell death protein 1), CTLA-4 (cytotoxic T lymphocyte-associated antigen-4) and BTLA (B- and T-lymphocyte attenuator), and induced apoptosis of T cells through activation of Fas/Fas ligand pathway (94). Furthermore, the poor prognosis of B-cell lymphoma patients with exosomal BCL6 and *MYC* mRNA was observed at diagnosis (97).

#### 3.2.5 miR-150 in MALT

Mucosa-associated lymphoid tissue lymphoma (MALT lymphoma) is a type of B-cell NHL characterized by monoclonal B-cell infiltration. Most MALT lymphomas occur in organs without lymphoid tissues, such as the stomach, orbits, intestines, skin, lungs, thyroid, parotid glands, soft tissues, bladder, kidneys, and the central nervous system. Studies have shown that the expression of MIR-150 in MALT lymphoma tissues is upregulated to various degrees (76–78), suggesting miR-150 is a potential tumor marker for monitoring MALT lymphoma. In the study of conjunctival MALT lymphoma, highly expressed MIR-150 inhibited the expression of casitas B-lineage lymphoma proto-oncogene b (*Cbl-b*) (HGNC:1542), an E3 ubiquitin linker, promoting the proliferation, migration, and invasion of lymphoma cells and inhibiting apoptosis (76). In gastric MALT lymphoma, the upregulated MIR-150 inhibited apoptosis and induced B cell proliferation by inhibiting the expression of early growth response 2 (*EGR2*) (HGNC:3239) of the B cells pro-apoptotic genes (53, 77, 78).

#### 3.2.6 miR-150 in MCL

MCL is a B-cell NHL with a high degree of malignancy. Studies have found that MIR-150 expression in MCL is significantly reduced (54, 56, 79); however, the mechanism by which miR-150 regulates B cells in MCL remains to be explored. Moreover, a study shows that the cell-specific uptake of MCL exosomes by normal and MCL patients’ B-lymphocytes is in a lipid raft-dependent manner, and MCL-derived exosomes preferentially internalized into B-lymphocytes subsets (98).

### 3.3 Regulation of miR-150 in autoimmune diseases

Previous studies showed that miR-150 might be a critical regulator of gene expression during immune cell differentiation and immune responses. Its regulatory function in the cellular immune process contributes to host defense against invading pathogens. Dysregulated expression of MIR-150 in immune cells may result in autoimmune diseases (99). MIR-150 is upregulated or downregulated in many autoimmune diseases, such as multiple sclerosis (MS) (100), myasthenia gravis (MG) (101–103), systemic lupus erythematosus (SLE) (104–106), rheumatoid arthritis (RA) (107–109), and primary Sjogren’s syndrome (pSS) (110). Studies have indicated that increased B cell activity and autoantibody production are hallmarks of autoimmune diseases such as MS (111), MG (103), SLE (112, 113), RA (114), and pSS (110). In the following section, we will summarize the potential role of miR-150 on B cells and discuss the potential role of miR-150 in the pathogenesis of autoimmune diseases (**Table 2**).

**Table 2:** Regulation of miR-150 in autoimmune diseases.

Diseases	miR-150	Samples	Effection	Clinical outcome	Reference
MS	Cycling miR-150↑	CSF	B cell percentage↑	Aggravate diseases	(115)
	Evs miR-150↑	Serum	Cognitively impaired	Aggravate diseases	(116)
MG	miR-150↑	Thymic GC B cells	Tregs dysfunction	Aggravate diseases	(103)
	Exosomal miR-150↑	Serum	CD19^+^ and CD27^+^ B cell↑	Aggravate diseases	(117)
SLE	miR-150↑	DN B cell	Autoantibodies↑	Aggravate diseases	(110, 118, 119)
	miR-150↓	B1a cell	B1a cell proliferation↑ IgM↑	Aggravate diseases	(25)
	Cycling miR-150*	Plasma	NA	NA	(120–122)
	Exosomal miR-150↑	Urine	Renal fibrosis↑	Aggravate diseases	(123)
RA	miR-150↑	PBMCs	NA	Aggravate diseases	(108, 124)
	miR-150↓	Human Synovial Tissue	MMP14↑ VEGF↑	Relieve diseases	(107)
pSS	miR-150↓	PBMCs	Autoantibodies↑	Aggravate diseases	(110)
AIHA/ES	miR-150↓	B cell	Autoantibodies↑	Aggravate diseases	(125)

SLE, systemic lupus erythematosus; RA, rheumatoid arthritis; pSS, primary Sjogren’s syndrome; MS, multiple sclerosis; MG, myasthenia gravis; DN B cell, IgD^-^CD27^-^ double negative B cell; PBMCs, peripheral blood mononuclear cells; CSF, cerebrospinal fluid; Evs, extracellular vesicles; GCs, germinal centers; Tregs, regulatory T cells; MMP14, matrix metalloproteinase; VEGF, vascular endothelial growth factor; NA, Not available. ↑, increase; ↓, decrease.

*The studies showed conflicting results in SLE and LN.

#### 3.3.1 miR-150 in MS

MS is a complex inflammatory demyelinating disease of the central nervous system. It is one of the most common causes of neurological disability in young adults. Accumulated studies showed that B cells play a critical role in MS pathogenesis (126). B cells participate in the pathophysiological changes of MS in various ways (127), including cytokine secretion and autoantibody production promoting a humoral immune response through the activation of complementary and antibody-dependent cytotoxicity. B cells participate in MS pathogenesis as dedicated antigen-presenting cells, amplifying autoimmune T cell responses leading to an inflammatory cascade of demyelination and nerve damage. Our recent research also demonstrated that suppressing the production of CD19^+^B cells can ameliorate MS in an experimental allergic encephalitis (EAE) mouse model (128). Another study showed that circulating miR-150 levels are elevated in the cell-free cerebrospinal fluid (CSF) of MS patients (115), and correlated with the clinical activity of the disease. It is widely acknowledged that enrichment of intrathecal oligoclonal bands (OCBs), the products of clonally expanded B cells in the CSF, is the most characteristic feature of MS (129). They also identified that OCBs positive patients had higher miR-150 than OCB-negative patients, indicating that miR-150 may associate with the products of clonally expanded B cells within the CSF (130). In our previous study within an EAE model, deletion of MIR-150 alleviates central demyelination and axon damage and upregulates the number of splenic CD19^+^B cells (100). The recent research shows that myeloid extracellular vesicles (Evs) from cognitively impaired MS patients expressed higher levels of MIR-150 compared to cognitively preserved MS patients (116). Therefore, we speculate that miR-150 is one of the modulators of altered B cell activity and plays multiple roles in the pathogenesis of MS.

#### 3.3.2 miR-150 in MG

MG is a T cell-dependent chronic autoimmune neuromuscular disease. 85% of patients suffer from muscle weakness due to anti-acetylcholine receptor (AChR) antibodies at the neuromuscular junction (103, 131). The main feature is ectopic B cell infiltration leading to thymus hyperplasia (132–135). The number of regulatory T cells (Tregs) in the thymus of MG patients is normal but dysfunctional (136). Hence, Tregs are unable to control the autoimmune response and prevent autoimmunity. Interestingly, miR-150 is particularly relevant to the T cell maturation process (6, 137). A study by Punga (101) showed that circulating miR-150 in the serum of patients with early-onset MG was significantly upregulated, and the expression was reduced after thymectomy, accompanied by improvement in symptoms. Another study of patients with advanced MG showed a negative correlation between the expression of circulating miR-150 and an improvement in the patients’ clinical conditions (102). Cron et al. (103) observed an upregulation of MIR-150 in the MG thymus associated with the presence of thymic B cells. *In situ* hybridization experiments showed that miR-150 was primarily expressed in the epithelial region of thymus GCs. They also showed that the high level of miR-150 secreted by B cells in thymic ectopic GCs affects the development of T cells by locally inhibiting the expression of *MYB*, resulting in T cell dysfunction and ultimately promoting the occurrence and development of MG. Zhong et al. (117) demonstrate that serum exosomal miR-150 decreased after low-dose rituximab (RTX) treatment in patients with anti-AChR antibodies positive refractory MG, and alleviated symptoms of MG, these results suggest that the relationship may be related to miR-150 interactions with CD19^+^ and CD27^+^ B cells.

#### 3.3.3 miR-150 in SLE

SLE is a heterogeneous autoimmune disease. The production and development of the disease are closely related to B cells. Studies have found that the severity of SLE and lupus nephritis (LN) is related to circulating miR-150 in plasma. Several researches showed that circulating miR-150 levels in SLE and LN patients were reduced compared to healthy controls (120). In contrast, some studies found that the expression levels of circulating miR-150 were upregulated in the plasma of patients with SLE and stage III LN (121, 122). Sole et al. demonstrated that miR-150, which promotes renal fibrosis by downregulation of SOCS1 (antifibrotic protein suppressor of cytokine signaling 1) (104), is significantly overexpressed in urinary exosomes from patients with LN compared to healthy controls, and MIR-150 expression levels increase progressively according to the degree of LN chronicity index (CI), being most highly expressed in the high CI group (123). The conflicting results of these studies may be due to the limited number of samples studied. Also, the level of circulating miR-150 may be related to the different disease courses of SLE and LN.

Chen et al. (110) showed that the expression of MIR-150 in CD19^+^IgD^-^CD27^-^ double negative (DN) B cell subsets were upregulated in SLE patients compared with healthy controls, and the levels were positively correlated with the percentage of DN B cells and plasmablasts. Previously, miR-150 was considered a sensor for general lymphocyte activation induced by inflammation (138), and that DN B cells can migrate into inflammatory tissue (118, 119), leading to an increase in the number of autoantibodies; thus, promoting the development of disease. Jiang et al. (25) showed that the percentage of FLT3^+^ B1 cells in SLE patients was significantly higher than that of healthy controls, while the MYSM1 levels in FLT3^+^ B1 cells from these patients decreased. The surface IgM level was positively correlated with the percentage of FLT3^+^ B1 cells. Further research showed that the level of miR-150 in circulating FLT3^+^ B cells in SLE patients was lower than that of FLT3^-^ B cells. It showed that the MYSM1/miR-150/FLT3 pathway that inhibits B1a cell proliferation is defective in SLE patients. This indicates that the reduction of miR-150 in the B1 cells of SLE patients leads to an increase in the proportion of FLT3^+^ B cells. The latter promotes the proliferation of B1a and the increase of surface IgM levels, and promotes the disease course of SLE.

#### 3.3.4 miR-150 in RA

RA is a chronic autoimmune disease characterized by the infiltration of leukocytes into joints, causing the production of inflammatory mediators and the destruction of cartilage and bone (139). Rezaeepoor et al. (124) showed that miR-150 was significantly increased in PBMCs in RA patients compared to the healthy control group. The level of miR-150 in the synovial samples of patients with RA synovitis is positively correlated with the severity of the joint destruction and the high activity of the disease (108). In addition, MIR-150 is significantly upregulated during interleukin 17 (IL-17) cell differentiation (108), which is important for the pathogenesis of RA (140, 141). Moreover, MIR-150 is downregulated and matrix metalloproteinase 14 (MMP14) while vascular endothelial growth factor (VEGF) are upregulated in RA, and mesenchymal stem cell derived miR-150 exosomes inhibit RA fibroblast-like synoviocytes migration and invasion, as well as suppressing angiogenesis by downregulation of MMP14 and VEGF, as a result, the symptoms of RA alleviate (107). Studies have pointed out that excessive activation of B cells is key to promoting the progression of RA (142, 143). However, whether miR-150 is involved in the regulation of B cell function in RA is still unknown.

#### 3.3.5 miR-150 in pSS

The latest research shows that MIR-150 expression is significantly down-regulated in pSS, and the percentage of DN B cells is also reduced. This is probably due to the insufficient expression of MIR-150, which leads to B cell differentiation and activation, and promotes the production of specific self-antibodies, leading to the development of pSS specific autoimmune processes (110).

#### 3.3.6 miR-150 in AIHA/ES

Autoimmune hemolytic anemia (AIHA) and Evans syndrome (ES) are two forms of B cell-mediated autoimmune cytopenia. B lymphocytes synthesize and secrete autoantibodies and play crucial roles in the pathogenesis of AIHA/ES. The study by Xing (125) reveals that the level of miR-150 in B lymphocytes from AIHA/ES hemolysis patients’ peripheral blood is significantly lower than that in the healthy controls, and it partially reveals the severity of AIHA/ES, because of the inverse correlation with total bilirubin (TBIL) concentration and indirect bilirubin (IBIL) concentration and positive relation with the complement 3 (C3) level. This study also shows that MYB level in B lymphocytes in the AIHA/ES group is much higher than that in the remission group and healthy controls. The expression of *MYB* was negatively related to hemoglobin and C3 and positively related to TBIL and IBIL. These results indicate that the level of miR-150 and MYB in B cell partially reveal the severity of the disease and the immune ability. An early study (144) pointed that miR-150 negatively regulated the endogenous *MYB* gene at both mRNA and protein levels. It can be speculated that miR-150 regulates B lymphocytes in AIHA/Evans syndrome through *MYB*, and the underlying mechanism needs further study.

### 3.4 ceRNA network of lncRNA/miR150 in B lymphocyte-related diseases

Competing endogenous RNA (ceRNA) is a multi-hub network consisting of long non-coding RNA (lncRNA), miRNA, and mRNA, where lncRNAs act as endogenous molecular sponges of miRNAs to regulate the expression of mRNAs (145). Its regulatory mechanism is involved in carcinoma initiation, progression and invasion (146–148). LncRNA/miRNA-associated ceRNA can work as circulating prognostic biomarkers offering prognostic value in B lymphocyte-related diseases. Mara et al. (149) have been constructed an intricate network comprising *Metastasis Associated Lung Adenocarcinoma Transcript 1* (*MALAT1*) (HGNC:29665)*-Enhancer Of Zeste 2 Polycomb Repressive Complex 2 Subunit*) *EZH2* (HGNC:3527)*-MYC-*MIR-150*-MYB* to explore the development progression and prognosis of non-Hodgkin lymphomas (NHL) (150). *MALTA1* is involved in both somatic hypermutation and class-switch recombination in B cell activation (151). Overexpressed *MALAT1* interacts with *EZH2* which is highly expressed in B-cell lymphomas, facilitating the complex binding to MIR-150 promoter region which increases *MYC* expression (152, 153). Moreover, *MALAT1* codes a retained lncRNA which sponges miR-150 and unleashes *MYB* from miR-150-mediated repression, promoting lymphoma cells proliferation and inhibiting apoptosis (149, 154, 155). Few studies on the ceRNA network involve miR-150 in B cell-related disorders. This is worth exploring in depth.

## 4 Conclusion and perspectives

As a core in the regulatory network, miR-150 is highly expressed in B cells, playing a key role in B cell development, proliferation, differentiation, and survival. In malignant tumors related to B cells, miR-150 controls different signal axes by regulating the multiple genes that affect the proliferation, differentiation, metabolism, and apoptosis of malignant B cells, ultimately affecting the invasion and progression of the tumor. However, many questions remain to be addressed.

Firstly, miR-150 affects the growth and development of B cells and immune response by inhibiting the target genes *MYB, FOXP1, FLT3, survivin*, and BCR signaling. However, it is important to explore how miR-150 regulates the sensitivity of autoreactive B cells to BCR stimulation: locating target gene in self-reactive B cells and enhancing the sensitivity to BCR stimulation. Furthermore, miR-150 was shown to be a biomarker of various autoimmune diseases. However, how abnormally expressed MIR-150 regulates the function of B cells and controls the development of the disease need to be further studied. With advanced techniques such as transcriptomics and gene editing, it would be possible to unfold autoimmune disease pathogenesis of miR-150 and identify new targets for therapeutic intervention.

Secondly, ATB-derived miR-150 is to regulate the insulin resistance of adipose tissue and immune homeostasis, especially its expression level and roles in ATB cells regarding brown, beige, and white adipose tissue need to be investigated. Moreover, the regulation of miR-150 on normal B cell metabolism is uncertain. Besides, whether downregulation of MIR-150 by inducing the *SLC2A1*/GLUT-1 axis adjust glucose metabolism in B-cell lymphoma evokes new directions for the mechanistic understanding and treatment of B-cell malignant tumors. By using metabolomics and proteomics techniques, it is possible to identify specific metabolites and molecules that are crucial for B cell function. Furthermore, the impact of particular interventions on B cell metabolism could be investigated using animal models and cell culture methods. Selectively targeting important enzymes or chemicals involved in B cell metabolism, may help us comprehend the role of intracellular metabolism in B cell-related disease.

Thirdly, the exosome-associated miR-150 derived from specific lymphocyte subsets could help confirm whether circulating miR-150 activates specific B cell subsets occurring at distant sites. These lines of inquiry should benefit the assessment of pathogenic immune response during the course of cancers, auto-immune diseases and their treatments, as well as significantly contributing to the close, prompt monitoring of clinical trials with novel immune-regulatory medications, particularly in the early stages of clinical development. Understanding the impact of miR-150 in B cell-related disorders would provide new avenues for targeted therapies.

## Author contributions

Z-LH developed the concepts, acquired funding and revised the manuscript. Y-ZH contributed to concept development, literature review and writing. QL and P-FW was responsible for all images. X-PL collected and summarized data. All authors contributed to the article and approved the submitted version.
